# Fibroblast Growth Factor Signalling in the Diseased Nervous System

**DOI:** 10.1007/s12035-021-02367-0

**Published:** 2021-04-15

**Authors:** Lars Klimaschewski, Peter Claus

**Affiliations:** 1 Department of Anatomy, Histology and Embryology, Institute of Neuroanatomy, Medical University of Innsbruck, Innsbruck, Austria; 2 Institute of Neuroanatomy and Cell Biology, Hannover Medical School, Hannover, Germany; 3 Center for Systems Neuroscience, Hannover, Germany

**Keywords:** FGF, FGFR, Receptor, CNS, Brain, Spinal cord, PNS, Nerve, Ganglia, Regeneration, Degeneration, Remyelination, Demyelination, Glioma, Anxiety, Depression, Schizophrenia

## Abstract

Fibroblast growth factors (FGFs) act as key signalling molecules in brain development, maintenance, and repair. They influence the intricate relationship between myelinating cells and axons as well as the association of astrocytic and microglial processes with neuronal perikarya and synapses. Advances in molecular genetics and imaging techniques have allowed novel insights into FGF signalling in recent years. Conditional mouse mutants have revealed the functional significance of neuronal and glial FGF receptors, not only in tissue protection, axon regeneration, and glial proliferation but also in instant behavioural changes. This review provides a summary of recent findings regarding the role of FGFs and their receptors in the nervous system and in the pathogenesis of major neurological and psychiatric disorders.

## Introduction

### The FGF and FGF Receptor Families

Fibroblast growth factors (FGFs) comprise a large family of polypeptides. They are expressed in nearly all organisms, ranging from nematodes to vertebrates. The 22 members of the FGF family are highly conserved in gene structure and amino acid sequence. Several of these factors are secreted and implicated in differentiation and migration during organ development. Ten of them are expressed in the brain [1]. FGF3, FGF8, FGF15, FGF17, and FGF18 play key roles in early development by imparting positional information and regulation of gene expression involved in brain patterning [2]. At the adult stage, FGFs primarily act as homeostatic factors in tissue repair and cellular proliferation. Several FGFs and their receptors (listed in Table 1) have been demonstrated to be involved in the pathogenesis of neurological disorders including Parkinson’s and Alzheimer’s [3, 4] and will be the focus of this review. The experimental data presented here were mainly obtained in rats and mice, the leading model organisms used in biomedical research. Very few aspects were validated in humans, and those are included as well.

**Table 1:** Summary of confirmed expression and functional significance of the most relevant FGFs and FGFRs in the nervous system (+ indicates presence or positive effect, - indicates absence or negative effect; see text for references)

FGF	−1	−2	−7	−8	−9	−20	−22	R1	R2	R3
Absolutely required for CNS development				+				+	+	
Abundance in the adult nervous system	+	+						+	+	+
Lack of signal peptide	+	+			+	+				
Nuclear localisation	+	+						+		
Expressed in neurons	+	+	+	+	+	+	+	+	+	+
Expressed in astrocytes	+	+			+			+	+	+
Expressed in oligodendrocytes	+	+			+			+	+	+
Expressed in microglia	+	+						+		+
Schwann cell proliferation		+						+	+	
Astrocyte proliferation	+	+						+	+	
Glioma proliferation	+	+						+	(−)	+
Enhanced ECM production		+								
Neurogenesis		+		+		+		+	+	
Excitatory synapse formation							+	+	+	
Inhibitory synapse formation			+						+	
Stimulation of LTP	+	+						+		
Neuronal survival	+	+	+	+		+		+		–
Up-regulation after peripheral nerve lesion	+	+	+							+
Promotion of axonal regeneration in the PNS	+	+						+		
Axonal elongation (in pre-lesioned neurons)	+	+						+		
Axonal branching (in naive neurons)	+	+						+		
Interaction with myelin inhibitory signalling	+	+						+		
Noci- and thermoception		+	+					+	+	
Seizure induction	–	+	–				+			

FGF1 and FGF2 (acidic and basic FGF) are the most widely studied members of the FGF family expressed in neurons and glial cells [5]. An unusual feature of those ligands is the lack of a conventional signal sequence for export out of the cell and their exit via non-canonical mechanisms. Other FGFs like FGF11-14 remain intracellular and exert intracrine functions [6]. Some translocate from early endosomes into the cytosol and enter the nucleus [7]. This complexity is further enhanced by the expression of isoforms. For example, human FGF2 is expressed in five different isoforms derived from a single mRNA species [8]. This is the result of N-terminal sequence extensions in higher molecular weight isoforms as compared with low molecular weight FGF2 in some species [9, 10]. While the intranuclear functions of FGF1 and FGF2 are not fully understood [11], signalling through membrane-bound tyrosine kinase receptors has been described in detail [12]. Different FGF subfamilies exhibit preferences for one of the FGF receptors (FGFRs). FGF1 is the only member that can activate all four FGFR variants.

FGFRs share 46% amino acid identity and code for receptors of 125-160 kDa molecular weight. A fifth FGFR, FGFRL1, lacks the tyrosine kinase domain and is a putative co-receptor for FGFR1 [13]. FGFR1-3 are characterised by three extracellular immunoglobulin (Ig)-like domains, a heparin-binding region, and an acidic box domain. Ligand binding to D2 and D3 domains results in a 2:2:2 ternary complex of FGF, FGFR, and heparan sulphate [14]. The Ig-like D1 domain and the acidic box, located between D1 and D2, inhibit ligand binding by electrostatic interactions [15]. Alternative splicing in the D3 domain generates the IIIb and IIIc isoforms of FGFR1-3 with different ligand-binding properties (FGFR1b, -1c, -2b, -2c, -3b, and -3c). The b and c isoforms are restricted mainly to epithelial and mesenchymal tissues, respectively [16]. The extracellular D2 domain interacts with heparan sulphate proteoglycans (HSPGs) that facilitate the dimerisation and stabilisation of ligand interactions. The intracellular region of FGFRs beneath the transmembrane segment harbours a split tyrosine kinase domain. The binding of FGFs at HSPGs allows the formation of defined ligand gradients required for paracrine signalling, in particular during development.

### Nuclear FGFR Signalling

In addition to its canonical role as membrane-bound tyrosine kinase receptor, FGFR1 has been described as a nuclear protein [17]. It translocates to the nucleus via an importin-β-dependent mechanism [18]. On the functional level, the nuclear receptor is a major signalling hub (designated as nuclear FGFR1 signalling, INFS) regulating neuronal growth and differentiation, amongst others [11, 19, 20]. Nuclear FGFR1 colocalises with transcriptionally active chromatin, binds to CREB-binding protein (CBP) or ribosomal S6 kinase isoform 1 (RSK1), and forms complexes with retinoid and Nurr receptors. Developmental signals are thereby directly forwarded to CBP and RSK1. RSK1 binding promotes FGFR1 release from the pre-Golgi to the cytosol, increases the mobile population of FGFR1, and facilitates nuclear accumulation. Novel interactive features of FGFR1 allow the newly synthesised 90 kDa protein to be released from pre-Golgi membranes and translocate into the cell nucleus along with the nuclear localisation signal (NLS)-containing FGF2 ligand [21]. Granzyme B-dependent cleavage of the C-terminal part of FGFR1 may also play a role [22]. The mRNAs for FGF2 and tyrosine hydroxylase are up-regulated in response to the nuclear shuttling of the receptor [23]. Importantly, nerve growth factor (NGF) utilises INFS for its neurodevelopmental and gene-activating functions [24]. NGF induces process outgrowth and transcriptional programming in a neuronal cell line (PC12) via nuclear translocation of FGFR1. FGFR1 interacts with the orphan nuclear receptor Nurr1, and this complex regulates tyrosine hydroxylase (TH) expression by binding to the TH gene promoter [25]. Furthermore, INFS appears to be necessary for dendritic outgrowth of sympathetic neurons in response to bone morphogenetic protein, BMP7 [26]. Recent evidence suggests a broader function of nuclear FGFR1 on several genes relevant in nuclear development [21, 27].

### Distribution of FGF Receptors in the Nervous System

FGFR1-3 are widely distributed in the brain and bind to FGF ligands with different affinities and specificities [28, 29]. FGFR4 plays a role in early brain development but is absent from the adult brain (apart from one small nucleus). FGFR1 is most abundant in the nervous system with a predominant expression in neurons, astrocytes, and radial glia [30]. FGFR2 and FGFR3 are preferentially expressed in astrocytes and oligodendrocytes [1]. Studies of mice carrying null mutations in each of the FGFR genes revealed that FGFR1 and FGFR2 are essential for early embryonic development, which reflects their key roles in neuralisation and precursor proliferation. In contrast, animals lacking FGFR3 survive and exhibit no obvious telencephalic defects. However, FGFR3 plays an important role in cortex development [3]. Mutations in FGFR2 lead to either Apert or Crouzon syndrome, resulting in prominent changes of several brain structures [31].

The overlapping pattern of FGF ligand binding to similar FGF receptors implies a certain level of redundancy. In fact, different FGF family members activate FGFR subtypes to different degrees, depending on their ability to bind with high or low affinity to each receptor subtype [32]. Moreover, receptor specificity is modulated by the expression of other receptors and by the specific lipid composition of the plasma membrane. For example, in oligodendrocytes, a fraction of FGFR2 resides within the cholesterol/glycosphingolipid-enriched membrane microdomains (lipid rafts) [33]. Lipid rafts concentrate and segregate surface receptors together with their signalling molecules, and this compartmentalises and enhances intracellular signal transduction [34].

The complexity of FGFR activation is further increased by membrane molecules that may directly bind to and activate FGFRs in the absence of canonical ligands. Some of them play a pivotal role in the nervous system. For example, neural cell adhesion molecule (N-CAM), neuronal cadherin (N-cadherin), Eph receptor A4 (EphA4), and Anosmin-1 use FGFR as a signalling mediator and interfere with intracellular FGFR transport [35–37]. Hence, FGFRs are not specific to FGFs, and the phenotype of mice deficient in one or more FGFRs may be due to the lack of FGFR activation by ligands unrelated to FGFs or other receptors.

### Signal Transduction of FGF Receptors

**Fig. 1:**
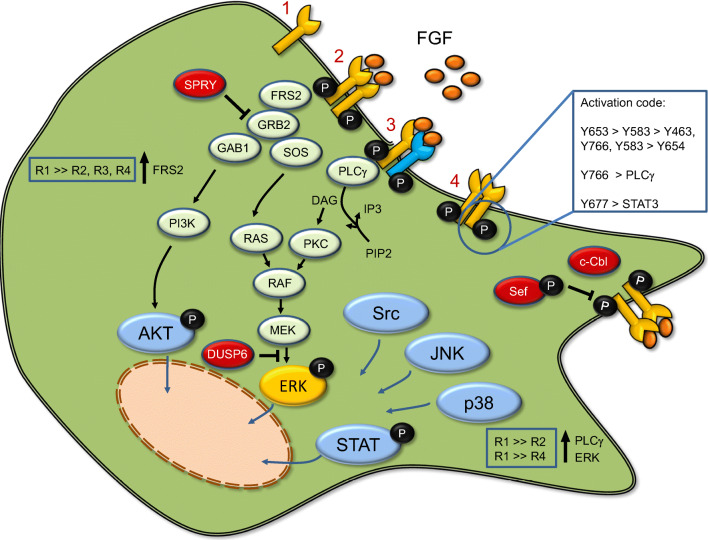
The neuronal FGFR signalling network, from the binding of ligands to downstream events. FGFR1-3 monomers (1) form homodimers (2) and heterodimers (3) either in a ligand-dependent (2, 3) or independent (4) mode. The latter may undergo autophosphorylation. FGF ligand binding leads to enhanced receptor phosphorylation. The ordered and cooperative post-translational modifications are depicted as an activation code with sequentially phosphorylated tyrosine residues (boxed inset, 4). Certain downstream pathways like PLCγ and STAT3 require specific phosphorylation of additional tyrosine residues. Phosphorylation activates downstream pathways such as PLCγ, AKT, ERK, and STAT3. These are regulated by a number of proteins that provide an inhibitory feedback, thereby limiting activation (SPRY, Sef, and DUSP6; depicted in red). The lipid-anchored fibroblast growth factor receptor substrate 2 (FRS2) undergoes phosphorylation by FGFR kinase activity and recruits downstream factors PI3K and Ras/ERK as signalling hubs. However, this mechanism is FGFR-specific, with FGFR1 showing higher activity than the other FGFRs (boxed inset). Moreover, FGFR subtypes differentially activate downstream ERK and PLCγ with FGFR1 showing stronger activation than FGFR2 or FGFR4, respectively (boxed inset). For references, see text

Ligand binding in cooperation with accessory HSPG triggers dimerisation of receptor monomers. This results in their mutual activation [38]. All possible FGFR combinations formed amongst FGFR1-3 suggest that FGFR heterodimers are as functionally important as homodimers [39]. FGFR dimers may also form in the absence of ligands at their physiological concentrations (Fig. 1). The ligand-independent dimers are stabilised through contacts below the transmembrane domains. These receptors are auto-phosphorylated, which explains why FGFR overexpression can lead to certain forms of cancer. The primary effect of ligand binding lies in a structural change in the pre-formed dimers and thereby enhanced receptor phosphorylation. Diverse ligands change receptor kinase activities in different ways. For example, FGF2-bound dimers show the smallest separation between the transmembrane domains but the highest possible phosphorylation [40].

Phosphorylated FGFRs activate canonical scr-homology 2 (SH2)-linked signalling proteins (PLCγ, CRKL) and recruit adapter proteins for connecting the receptor to PI3K/MAPK pathways. The analysis of chimeric receptors composed of cytoplasmic FGFRs and extracellular PDGF receptors has revealed that all FGFR subtypes stimulate the same pathways but with different magnitudes [38]. Prominent differences are found between FGFR1/FGFR2 and FGFR3/FGFR4 signalling. This may be due to differences in ligand activation and/or intracellular receptor transport following internalisation. For example, FGFR1 activates ERK and PLCγ more strongly than FGFR4, and higher ERK activation is caused by FGFR1 rather than by FGFR2 [16].

Key signalling hubs such as PI3K and Ras/ERK are recruited by the lipid-anchored 80 kDa docking protein FRS2 (also referred to as SNT1). FRS2 constitutively binds the juxtamembrane region of FGFR1 and is phosphorylated most efficiently by FGFR1 as compared with other receptor isoforms [41]. Activated FRS2α associates with the Grb2/SOS complex to relay activation of Ras and downstream MAPK signalling [42]. Additionally, FRS2α recruits the tyrosine phosphatase SHP2 [43]. FRS2α is also involved in neurotrophin receptor (Trk) signalling and appears to act as a ‘conning centre’ responsible for differential pathway activation. Furthermore, FRS2 is crucial for FGFR ubiquitination and trafficking due to its ability to constitute local signalling platforms and to recruit feedback inhibitors [44]. The latter initiate a cascade of negative signalling events that decrease the amplitude of positive signals and modulate the level of stimulation.

Six tyrosine (Y) residues in the split kinase domain need to be sequentially phosphorylated for the full activation of at least four major signalling pathways (Y653 → Y583 → Y463, Y766, and Y585 → Y654) [12]. Y653 increases tyrosine kinase activity by fifty- to one hundred-fold, and Y654 by a further ten-fold. Additional tyrosines are required for the activation of phospholipase Cγ (Y766) and STAT3 (Y677). The STAT pathway changes nuclear gene expression, whereas activation of PLCγ at the plasma membrane produces inositol trisphosphate (IP3) and diacylglycerol (DAG), thereby releasing calcium from endoplasmic reticulum stores and causing the activation of protein kinase C (PKC), respectively. DAG also produces ligands that can activate the endocannabinoid receptor CB1 in the brain [45]. Importantly, cannabinoid receptors transactivate FGFR1 in lipid rafts [46].

Inhibitory feedback mechanisms are induced by FGFR stimulation, which is essential to limit excessive signalling. Their de-regulation may result in brain tumours. They involve the coordinated action of ubiquitin ligase (c-Cbl), adapters (Grb2) and proteins such as Sef, phosphatases (DUSP), and Sprouty proteins [47, 48]. The latter function as crucial FGFR antagonists during brain development and in the adult, mainly by interfering with processes upstream of ERK [49]. Interestingly, FGFRs themselves are the subject of negative feedback mechanisms, because the prevention of ERK-dependent phosphorylation at serine 777 of FGFR1 (or the mutation of this serine to alanine) promotes receptor tyrosine phosphorylation and, consequently, cellular proliferation, migration, and axon growth [50].

### Intracellular FGF Receptor Transport

FGFR activation is followed by rapid endocytosis and degradation of the receptor and the ligand. Ligand binding induces receptor mono-ubiquitination by the ubiquitin ligase c-Cbl, which functions as a signal for the sorting of the receptor into intraluminal vesicles of multivesicular endosomes and its subsequent delivery to lysosomes [51]. Receptor tyrosine kinases, such as FGFR, EGFR, and PDGFR, are mono-ubiquitinylated at multiple sites, while cytoplasmatic phosphorylated protein tyrosine kinases are poly-ubiquitinylated and degraded in the proteasome [52]. In the case of FGFR signalling, c-Cbl does not directly bind to the receptor but catalyses the ubiquitination of the receptor via interaction with FRS2 and Grb2. Hence, the competition of c-Cbl with SOS for Grb2 abrogates MAPK signalling [53].

The transport of FGFRs from the cell surface to different subcellular compartments influences the biological response to receptor activation. This has recently been confirmed by optogenetics [54]. Overexpressed FGFR1-eGFP fusion proteins bind FGF2 and activate signalling hubs at various locations. FGFRs internalise and shuttle to the recycling and degradation compartments in neurons and glial cells. This has consequences for the strength and duration of signalling pathway activation [55, 56].

Plasma membrane levels of neuronal FGFRs at the adult stage appear to be significantly lower than those of neurotrophin receptors, because the effects of neurotrophins on neuronal survival and neurite outgrowth are significantly stronger when compared with FGFs. However, overexpression of FGFR1 stimulates axon growth [57]. This effect is further enhanced by protease inhibitors such as leupeptin, which inhibits lysosomal protein degradation and promotes receptor recycling [58].

RTK recycling depends on the number of intracellular lysine residues that are required for receptor ubiquitination. For example, FGFR1 comprises 29 lysine residues, while the intracellular part of FGFR4 harbours 16 lysine residues only. Accordingly, lysine mutants of FGFR1 that are deficient in ubiquitination will be sorted to the recycling pathway rather than to degradation in lysosomes [59]. Overexpression of FGFR1 mutants exhibiting reduced numbers of lysines modifies axon outgrowth [60]. FGFR1-15R (with 14 instead of 29 lysine residues) preferentially recycles back to the plasma membrane similarly to FGFR4 and strongly promotes elongative axon growth without stimulating axon branching. Interestingly, the ERK inhibitor PD98059 does not reduce elongative axon growth induced by FGFR1-15R overexpression. This raises the possibility that ERK has independent effects on the axonal cytoskeleton through enhanced receptor recycling of FGFR1 which probably shows increased interaction with other growth promoting membrane receptors (e.g., NCAM [61]). The functional significance of the Ras/RAF/ERK pathway for adult axon regeneration remains a subject of controversy because in some studies ERK inhibitors did not interfere with axon outgrowth of adult primary neurons in culture [62].

## FGFs in Neurological Disorders

### Neuronal Degeneration and Repair

As master regulators of brain organogenesis and homeostasis, FGFs play an important role in the regeneration and repair of the nervous system. In fact, FGFR1 and FGFR2 stimulation induces complete neural tissue regeneration in planarians and vertebrate embryos [63]. Moreover, FGFs often synergise with other growth factors and cytokines in the generation of multipotent progenitors, for example, in the zebrafish retina [64]. In adult mammalian species, however, FGFs cannot replace damaged tissue, although stimulation of FGFR signalling assists in adult neurogenesis [65] and promotes neuronal survival after injury [1]. Conversely, expression of dominant-negative FGFR results in increased neuronal vulnerability [66]. The neuroprotective functions of FGFs are at least partially mediated by direct stimulation of neuronal FGFRs and are related to the inhibition of autophagy/protein clearance in a PI3K/AKT/mTOR-dependent manner [67].

In addition to preventing or delaying neuronal cell death, FGFs are involved in the repair of synaptic connections. The formation of new excitatory synaptic contacts is regulated by FGF22, which is expressed in spinal interneurons and long propriospinal neurons [68]. In fact, a lack of FGF22 or targeted deletion of FGFR1 and FGFR2 in the motor cortex reduces synapse formation between corticospinal collaterals and relay neurons and attenuates functional recovery in response to spinal cord injury [69]. FGFR1b and 2b are required for excitatory and inhibitory presynaptic differentiation in response to FGF22 and FGF7, respectively [70, 71]. Both receptors mediate the excitatory presynaptic response to FGF22, whereas only FGFR2b elicits the inhibitory presynaptic response to FGF7.

FGF7-deficient mice exhibit epileptogenic changes in the hippocampus. This indicates that inhibitory synapse formation may be impaired, resulting in mossy fibre sprouting and enhanced neurogenesis during development [72]. Blocking FGF22 while activating FGF7 signalling may help to alleviate epileptogenesis. In general, it is assumed that FGFs are implicated in both seizure susceptibility and seizure-induced plasticity. It has been suggested that FGF2 favours acute seizures but reduces seizure-induced cell death [73, 74]. In the amyotrophic lateral sclerosis (ALS) model of mutant SOD1 mice, FGF deficiency causes a significant delay in disease onset, less impaired motor function, and prolonged survival when compared with mice with normal FGF2 levels, probably due to an up-regulation of neurotrophic factors such as CNTF and GDNF [75].

FGF2 and FGF20 synergise to increase dopaminergic neuron numbers in stem cell models [3]. FGF20 has been found to be preferentially expressed in the substantia nigra, pars compacta. It stimulates survival of dopaminergic neurons via activation of FGFR1IIIc [76]. In addition, FGF2 facilitates the formation of functional dopaminergic neurons from stem cells [77, 78]. In the 6-hydroxydopamine lesion model, infusion of FGF20 into the substantia nigra protects against cell death in both the substantia nigra and striatum, and this is accompanied by improved motor function [79]. Moreover, intrastriatal expression of FGF2 results in dopaminergic neuron recovery following chemically induced lesions [80].

With regard to Alzheimer’s disease, overexpression of FGF2 restores spatial learning, long-term potentiation, and neurogenesis. These effects are probably mediated by FGFR1-activated increases in CD200, the OX-2 membrane glycoprotein that regulates microglial activity and promotes neurite outgrowth and neuronal survival [81]. Furthermore, exogenous FGF2 ameliorates tau pathology and spatial memory deficits by down-regulating the amyloid precursor protein-cleaving enzyme (BACE1) that is involved in the production of amyloid β [82]. In primary hippocampal neuron culture, protection against amyloid β-induced neurotoxicity has been demonstrated to be dependent on the AKT but not the ERK signalling pathway. Interestingly, high molecular weight isoforms of FGF2 are more efficient than those of low molecular weight in this paradigm [83].

**Fig. 2:**
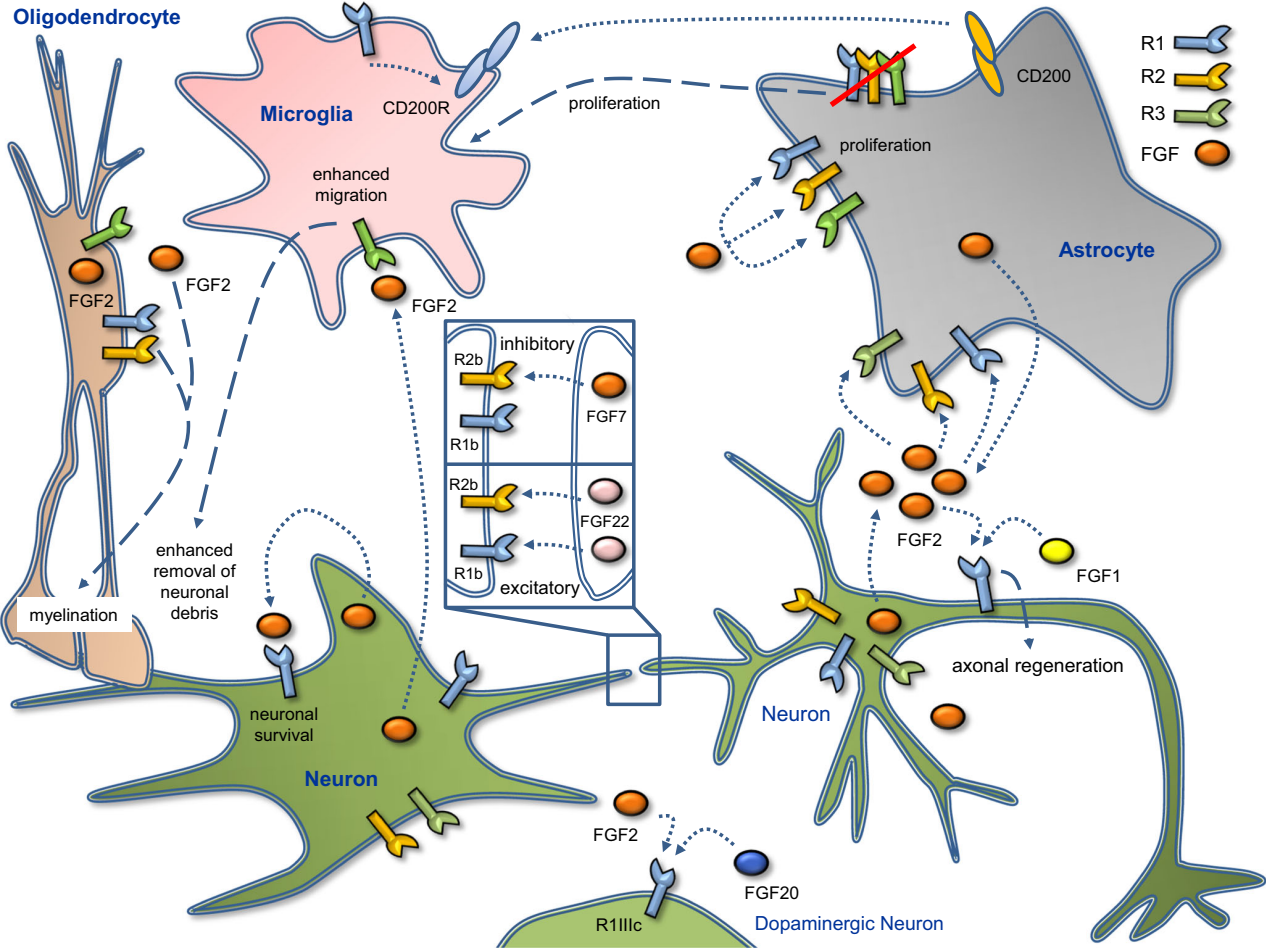
Key mechanisms of FGF/FGFRs in the nervous system. The central nervous system comprises a large number of functionally and structurally diverse neuronal and glial cell types. The figure depicts model neurons forming synaptic connections as well as oligodendrocytes, astrocytes, and microglia. Modulation of synaptic connections by FGFs (boxed inset): FGF22 regulates the formation of excitatory synapses together with FGFR1b and R2b. Although both are involved in excitatory synapse regulation, inhibitory synapses are regulated by FGF7 via FGFR2b only. FGF2 and 20 synergise to regulate differentiation of dopaminergic neurons by using FGFR1IIIc as the receptor (FGF20 also binds to FGFR1IIIc in other neurons). When secreted by neurons, FGF2 enhances microglia activation, leading to increased removal of neuronal debris in case of neuronal damage. Moreover, FGF2 restores spatial learning, long-term potentiation, and neurogenesis in Alzheimer´s disease. Mechanistically, FGFR1 regulates CD200, which in turn mediates microglia responses and neurite outgrowth. This factor also feeds back by activation of FGFR1. Axonal growth and regeneration is stimulated mainly by FGF1 and FGF2. FGF2 secreted by neurons stimulates astrocytes via FGFR1-3 activation. Signalling from astrocytes to oligodendrocytes is accomplished by FGF2 influencing the survival and proliferation of oligodendrocyte precursor cells (OPCs). FGFR1 and R2 regulate myelin thickness and gene expression. For references, see text

Endogenous FGF2 is secreted by neurons upon damage by glutamate or oligomeric amyloid β. This is followed by enhanced microglial migration and neuroprotection because of increased phagocytosis of neuronal debris via FGFR3 activation involving ERK and Wnt signalling [84]. However, loss of all three FGFRs in astrocytes results in microglia hypertrophy and proliferation [85]. These findings indicate a key role for FGF2 and FGFRs in orchestrating the crosstalk between degenerating neurons, microglia, and astrocytes. They also show that cellular activation and proliferation are distinct and that FGF-dependent processes are induced at different points after injury (Fig. 2).

Glutamate-mediated neuronal damage is observed in the hippocampus following temporal lobe epilepsy. This brain structure is highly dependent on FGFR1 signalling during development via FGF-mediated stimulation of hippocampal progenitor and stem cells [86]. Hippocampal deficits observed in patients with neurodegeneration, trauma, Alzheimer’s disease, and in normal ageing may therefore be counteracted by the incorporation of newly born neurons into existing networks. In fact, hippocampal neurogenesis has been observed to facilitate learning and memory in rodents. Adult neurogenesis in the human hippocampus is, however, a heavily debated issue. Recently, neurogenesis has been demonstrated to be limited to early development and childhood [87]. Other groups have observed ongoing neurogenesis in the hippocampus and a modest decline with age by applying improved strategies for the visualisation of neuronal precursor cells [88].

Stimulating neurogenesis through enhanced FGF signalling in the adult hippocampus may therefore be beneficial, particularly since neurogenesis decreases progressively in the brains of Alzheimer’s disease-affected patients. It is not yet clear, however, whether intrinsic precursor cell activity or changes in their environment determines such decline. Although FGFs promote the proliferation of cultured adult hippocampal precursor cells, their requirement for *in vivo* hippocampal neurogenesis in the adult and ageing brain still needs to be demonstrated. Elegant studies including conditional expression of mutated FGFRs have revealed that FGF signalling is clearly required for stem cell maintenance and increased neuron production [89–91]. Moreover, activated FGFR restores age-related decline in neurogenesis to a level found in young adult animals [92].

Neuronal degeneration is often associated with oedema formation and vascular pathology. These are particularly common in ageing and regularly affect hippocampal formation [73]. FGFs exert beneficial effects in some of these conditions, for example, in retinal cell swelling [93] and ischemic-reperfusion or hypoxic injury [94, 95]. FGF1 mixed into fibrin glue as a slow-release carrier reduces ischemia-induced focal brain infarction and attenuates functional deficits. Hippocampal and cortical neuron loss as well as microglial infiltration are also reduced. In addition, FGFs induce up-regulation of tight junction proteins via RhoA inhibition, thereby mitigating blood-brain barrier (BBB) breakdown and secondary brain injury [96].

### Trauma in the CNS

Adult axon regeneration across spinal cord injuries and into intact spinal cord tissue generally fails in all higher vertebrates. Only a few growth factors, amongst them FGF1 and FGF2, were shown to promote axonal growth and functional recovery in spinal cord injury (SCI) models [97, 98]. The observed beneficial effects have been attributed generally to an attenuation of astrogliosis, increased numbers of neuronal progenitors, and/or stimulation of bipolar astrocyte morphology, which result in glial bridge formation guiding regenerating axons across the lesion site [99]. In fact, astrocytes use FGF2 as an auto- and intracrine signal to promote proliferation and structural changes in glial cells via FGFR1 or FGFR2 signalling. This effect can be enhanced by exogenous FGF2 [100]. FGF2 treatment shortly after spinal cord hemisection results in a significant reduction of TNFα expression at the lesion site, gliosis, and monocyte/macrophage infiltration 2 weeks later. Levels of astrocyte-derived chondroitin sulphate proteoglycans (CSPGs) are also markedly decreased, and functional recovery significantly improved [101]. Interestingly, similar effects were observed in the spinal cord of injured mice lacking Spry4, an endogenous feedback inhibitor of FGF signalling [102].

Recent evidence suggests, however, that FGF signalling is also required for the re-establishment of the *non-reactive* state of astrocytes following the initial phase after CNS injury. After applying conditional genetic approaches to manipulate FGFRs specifically in adult astrocytes, strong activation was observed in the lesioned neocortex of FGFR1-3 triple knock-out mice [85]. Both FGF1 and FGF2 inhibit GFAP expression via FGFR3 signalling [103, 104]. Since the formation of astrocytic scars clearly inhibits axon regeneration in the CNS, the reversal of the active state in astrocytes (probably involving changes in heparin sulphatation [105]) is likely to contribute to the positive effects of FGF1 and FGF2 on functional recovery after axotomy of intrinsic spinal cord or peripheral axons projecting into the CNS [91, 106–108].

A recent study [109] provided convincing evidence for a reversal of the dogma that adult CNS axons do not regenerate over long distances, by overcoming three obstacles simultaneously. The authors demonstrated that enhancing the intrinsic neuronal growth machinery and providing a supportive extracellular matrix via stimulation of FGF signalling, together with the application of chemoattractive cues, results in robust and successful axon regrowth across at least one spinal segment after complete spinal cord injury in rodents. In this model, osteopontin, IGF1, and CNTF were applied before injury. Growth-supportive substrates were induced by a combination of EGF and FGF2, which increased astrocytic proliferation as well as laminin, collagen, and fibronectin production inside the lesions. Then, propriospinal axons were attracted with GDNF delivered from biomaterial depots. All of these steps must be performed in combination to stimulate axon regeneration significantly, i.e. by a factor of around 100. Treatment with FGFs alone did not support axonal growth through astrocyte scars and across the lesion core into spared neural tissue. The combined approach resulted in the formation of terminal synapses and the re-establishment of electrophysiological conduction capacity [109].

Other studies confirm that stimulation of FGFR1 signalling alone is not sufficient to promote axon regeneration in the CNS. Up-regulation of FGFR1 in neurons projecting into the corticospinal tract (CST) does not enhance axon outgrowth. Injection of AAV serotype 1 overexpressing FGFR1 in a rat model of unilateral pyramidotomy did not increase sprouting of intact contralateral CST axons with overexpressed FGFR1, nor was it accompanied by functional improvements over control AAV injected animals [100]. Overexpression of FGFR1 in cultured cerebellar granule neurons even resulted in decreased neurite outgrowth. It is possible that key adaptor proteins, such as FRS2, are sequestered away from neurotrophic receptors promoting strong axon outgrowth, such as TrkA [110]. It has been shown previously that FGF2 exerts inhibitory effects on neurite outgrowth of cerebellar neurons plated on cortical astrocytes [111]. In addition, FGF1 and FGF2 may even interact with inhibitory receptors, such as the Nogo-66 receptor 1 (NgR1) [112].

NgR1 is a member of the Nogo receptor family implicated in the binding of myelin inhibitors and chondroitin sulphate proteoglycans [113]. It is part of a multi-component receptor complex comprising Lingo-1, p75, or TROY, which induces activation of the small GTPase RhoA, a well-known pathway involved in growth cone collapse and neurite outgrowth inhibition [114]. Interestingly, FGF2-dependent neurotrophic effects such as neuronal differentiation of PC12 cells and axonal branching in cortical neuron cultures are fully blocked by the ectopic overexpression of NgR1. Direct interaction between the two receptors could not be demonstrated; however, high-affinity binding of FGF2 to NgR1 was observed, suggesting that FGF family members also act as ligands at completely unrelated receptors [112].

### Nerve Injury in the PNS

In the peripheral nervous system (PNS), axons do regenerate in the absence of exogenous growth factor support, provided that the proximal nerve stump containing the severed axons is connected to its distal counterpart. FGF1, FGF2, FGF7, and FGFR3 are all rapidly up-regulated in the lesioned nerve and in corresponding ganglia after axotomy [115, 116]. FGF2 prevents apoptosis of sensory neurons when applied directly to the transected sciatic nerve [117]. FGF1 and FGF2 have both been shown to improve nerve regeneration across a collagen-filled nerve conduit [118, 119]. In fact, FGF2 is one of the most promising growth factor with regard to clinically relevant muscle re-innervation, because it induces neurite elongation of motor axons similarly to GDNF [120].

Channels filled with Schwann cells overexpressing the high molecular weight (HMW) isoforms of FGF2 are particularly useful in promoting nerve regeneration [121, 122]. Low molecular weight (18kD) FGF2 released from transduced Schwann cells also accelerates regeneration and functional recovery when it is used to repair the transected sciatic nerve [123]. Because of their effect on the mitogenesis of mesoderm- and neuroectoderm-derived cells, it is assumed that FGFs support axonal regeneration mainly via increased proliferation of Schwann cells and enhanced angiogenesis [118]. Yet, direct trophic effects of FGF2 isoforms on primary neurons are observed as well. Nevertheless, adult sensory neurons must be sensitised before FGF treatment by prior axotomy *in vivo*. In response to such a ‘pre-conditioning’ lesion, i.e. a sciatic nerve transection 1 week before extraction of the lumbar ganglia, FGF2 isoforms stimulate axonal elongation preferably [124]. This effect can be completely blocked by SU5402, a specific FGFR antagonist, and it is mediated by ERK and PI3K activation. Naïve, untreated sensory neurons exhibit only a little FGFR1 and FGFR2 at their surface, suggesting that axotomy-induced receptor upregulation may be involved in the regenerative response.

Hence, FGF2 does not exert prominent effects on peripheral axon outgrowth if neurons have not been pre-lesioned. However, increasing FGFR1 levels by overexpression, inhibition of degradation, or promotion of receptor recycling all stimulate peripheral axon regeneration [55, 125]. Additional treatment with the protease inhibitor leupeptin further increases outgrowth [57, 58]. FGFR3 stimulation induces the opposite response, since FGFR3-deficient mice reveal reduced neuronal apoptosis in response to nerve transection [126]. Transgenic mice expressing high levels of FGF2 reveal faster axon regeneration, probably as a result of combined effects on Schwann cell proliferation, delayed myelination, and on axons directly [127]. Moreover, intramuscular injections of FGF2 increase the amplitude of compound muscle action potentials, wet muscle weight, and motor endplate density [128].

Enhanced FGF signalling has also been shown to be beneficial in response to facial nerve injury [129]. The already poor recovery of regenerating facial axons is further compromised in FGF2 knock-out mice. However, FGF2-deficient animals exhibit no difference in the number of regenerating axons in the sciatic nerve. In fact, faster recovery of mechanosensory (but not of motor) function following sciatic nerve crush was observed, suggesting compensatory mechanisms in the lesioned peripheral nervous system of global FGF2 knock-outs [130].

Our own investigations on FGFR signalling antagonists like Sprouty proteins corroborate the positive effects of FGFs on axon regeneration. Primary sensory or hippocampal neurons dissociated from Sprouty2 knock-out mice or transfected with shRNAs against Sprouty2 and Sprouty4 reveal significantly enhanced axon outgrowth [131–133]. Following sciatic nerve crush, more myelinated axons regenerate in heterozygous Sprouty2 knock-out mice, and this is accompanied by faster recovery of sensorimotor performance and increased expression of the regeneration-associated GAP-43 protein [133].

As stated above, FGFRs interact with other membrane components that may affect neuronal survival and even axon regeneration. For example, FGFR1 is required for neurite outgrowth stimulated by CAMs (PSA-NCAM, α7 integrin, and N-cadherin) in neurons and in neuron-like cell lines [61, 134–136]. NCAM/FGFR1 receptor complexes could be particularly relevant for motor axon regeneration *in trans:* FGFR1 is found at the plasma membrane of Schwann cells, and polysialylated (PSA)-NCAM (but not FGFR1) is localised at the membrane of elongating motor axons during the early phase of regeneration. This hypothesis is further supported by the demonstration of an increased interaction of FGFR1 and PSA-NCAM following FGF2 treatment [120]. Moreover, FGFR and neurotrophin receptor (Trk) signalling is co-dependent as well. Rat pheochromocytoma (PC12) cells expressing dominant negative FGFR exhibit reduced NGF-induced process formation and autophosphorylation of FGFRs. Selective FGFR inhibitors or oligonucleotides that interfere with receptor binding completely block neurite outgrowth induced by NGF in this cell line [137].

With regard to the possible interaction with inhibitory signalling pathways, previous studies by the Schwab group have suggested that the Nogo/NgR system is not only relevant for the CNS but for the PNS as well. Axonal regeneration and functional recovery are impaired following sciatic nerve crush in transgenic mice overexpressing Nogo-A in Schwann cells [138]. By contrast, sciatic nerve regeneration is enhanced in NgR1 knock-out mice (authors’ observation). Importantly, NgR1 is expressed in adult DRG sensory neurons and in motoneurons [139, 140]. However, there is no evidence of a potential cross-talk between NgR1/p75/RhoA and FGF signalling in the PNS yet, although FGF2 has been demonstrated to act as potent inhibitor of RhoA in primary neuron cultures [141].

With regard to lesion-induced neuropathic pain, FGF7 may play a role in injury-induced nociception. It is localised in the large dense-core vesicles (LDCVs) of small-diameter primary sensory neurons and may be transported to the dorsal spinal cord [142]. FGF7 increases the amplitude of excitatory post-synaptic current evoked by stimulating the sensory afferent fibres in spinal cord slices. Intrathecally applied FGF7 potentiates a formalin-induced acute nociceptive response, while it is diminished in FGF7 knock-out mice. Mice deficient in FGF2 or FGFR1/FGFR2 exhibit decreased thermal pain sensitivity accompanied by neuropathy of unmyelinated axons in the dorsal spinal cord [143, 144]. Furthermore, continuous intrathecal infusion of FGFR1 inhibitors reduces neuropathic pain-related behaviour in the partial sciatic nerve lesion model via inhibition of p38 MAPK [145].

### Demyelination in the CNS

In the CNS, oligodendrocytes are required for myelination during development and in demyelinating disease. FGFR1 expression in these cells increases as the lineage progresses from oligodendrocyte precursor cells (OPCs) to mature oligodendrocytes. FGF2 and FGFR2 are also found in terminally differentiated oligodendrocytes [146]. Astrocyte-derived FGF2 positively influences the survival and proliferation of OPCs [147]. Conditional ablation of FGFR1 and R2 leads to the down-regulation of myelin gene expression, reduced myelin thickness, and axonal degeneration as knock-out mice age [148, 149]. FGFR3 is expressed in OPCs as well; however, FGFR3-deficient mice exhibit no changes in OPC proliferation rates [150].

Demyelination causes severe neurological deficits that are partially reversed by the spontaneous remyelination of axons by oligodendrocytes. Although the CNS is isolated from the peripheral milieu by the blood-brain barrier, remyelination can be triggered by peripheral factors that leak into the CNS after injury, including FGFs. Various FGFs are elevated in autoimmune diseases of the brain, such as multiple sclerosis: FGF1 in remyelinated lesions, FGF2 in active lesions and in the cerebrospinal fluid, FGF9 in active demyelinated lesions, and FGF21 in activated microglia or macrophages [151–153]. Intraventricular delivery of FGF2 induces severe disruption of mature oligodendrocytes, a marked loss of myelin, and aberrant accumulation of immature oligodendrocytes with a premyelinating phenotype [154]. Interestingly, the relative concentrations of the extracellular matrix protein Anosmin-1 and FGF2 in human MS lesions appears to be important for OPC migration through their interactions with FGFR1. This may have consequences for remyelination of lesioned axons because Anosmin-1 inhibits the effects of FGF2 on cellular migration [155–157].

FGF9 (also termed glia activating factor) is expressed by neurons and glia [158]. Like FGF2, FGF9 suppresses myelin protein synthesis by differentiating OPCs [159]. In multiple sclerosis (MS) lesions, FGF9 has been demonstrated to act indirectly, i.e. via the initiation of a complex astrocytic response that compromises remyelination [151]. By contrast, circulating FGF21, a member of the endocrine FGF family (expressed in the pancreas), stimulates OPC proliferation through interactions with β-klotho, an essential co-receptor of endocrine FGFs, in lysophosphatidylcholine (LPC)-induced lesions [160]. OPCs express β-klotho, the inhibition of which prevents increased OPC proliferation and remyelination.

Another level of complexity is introduced by the action of FGF1 and FGF2 in acute versus chronic MS lesions. FGF2 and FGFR1 levels are higher in MS patients than in controls, and a difference between relapse patients with higher FGF2 levels and those in remission is observed as well [161]. FGFR1 and FGFR2 double mutant mice exhibit hypomyelination in the chronic cuprizone model, indicating that FGF signalling (presumably via PI3K/AKT) is necessary for remyelination [162]. However, other studies have found the opposite, i.e., increased numbers of oligodendrocytes and improved remyelination of cuprizone-induced lesions or less myelin and axonal loss in MOG_35-55_-induced EAE in mice lacking FGF2 or FGFR1 [163–167]. This discrepancy may be due to the complexity of FGF signalling in multiple responses to injury and stress.

In experimental autoimmune encephalomyelitis (EAE), a model for MS, overexpression of FGF2 leads to enhanced remyelination and reduced axonal damage [168, 169]. Conversely, in the MOG_35–55_-induced EAE model of MS, FGF2 deficiency results in a more severe disease course, increased infiltration of lymphocytes and macrophages, and reduced remyelination [170]. By contrast, viral-mediated FGF2 overexpression results in less disease severity via inhibition of lymphocyte/macrophage infiltration [168]. FGF2 treatment clearly interferes with inflammation by reducing macrophages, microglia, and CD8 T-cells and limiting CD44-mediated leukocyte migration [168, 171, 172].

### Brain Tumour Formation

FGFRs are commonly overexpressed in many types of cancer. High levels of FGFR1 are associated with better overall survival in peripheral nerve sheath sarcomas [173], while activating mutations in the FGFR1 kinase domain have been found in a subset of glioblastoma patients with poor prognosis [174]. FGFR2 is expressed at lower levels in high-grade gliomas, which correlates with higher proliferation and lower survival rates [175]. These grade IV malignant gliomas are amongst the most lethal human cancers, because they are resistant to neurosurgery, cytotoxic chemotherapy, and radiation [176]. Most patients present with primary glioblastoma multiforme associated with irregular signalling of epidermal growth factor (EGF) receptors or mutated PTEN (phosphatase and tensin homolog). Combined RAS/AKT signalling and PTEN deficiency have been shown to act as the main drivers of these tumours [177]. Regulators of ERK signalling, such as the Sprouty proteins, also play an important role in glioma proliferation [178].

High levels of FGF1 and FGF2 have been detected in glioma tissue relative to the normal brain [179]. Whereas expression of FGFR1 is low in normal white matter, its synthesis is dramatically increased in malignant astrocytic tumours [180]. Nuclear FGFR1 contributes to increased proliferation of glioma cells [19]. The blockade of FGF signalling by various means, amongst them FGF2 antibodies, siRNAs against FGFR1, or treatment with the FGFR/VEGFR inhibitor PD173074, reveals small but significant growth inhibitory effects in glioma cell lines [181, 182]. FGF inhibitors stimulate apoptosis, inhibit glioblastoma invasion, and suppress angiogenesis [183]. Down-regulation of FGF2 potentiates the effect of temozolomide (TMZ), an oral alkylating agent, by inhibiting proliferation and migration, blocking the cell cycle in G0/G1, and promoting apoptosis [184]. However, the efficient treatment of brain tumours with antibodies, siRNAs, or pharmacological inhibitors remains a challenge (see *Therapeutic Approaches* below).

Glioblastoma is also one of the most highly vascularised cancers. Therefore, inhibition of FGF activity, for example, by overexpressing dominant-negative FGFR1, may constitute a therapeutic strategy for disrupting angiogenesis-dependent signals required for glioma growth and invasion [185]. FGF2 promotes angiogenesis directly by activating the proliferation and migration of endothelial cells and indirectly by upregulating urokinase-type plasminogen activator, which also leads to cell migration [186]. Furthermore, secretion of FGF2 by glioma cells enhances the blood-brain barrier function of endothelial cells, which may contribute to drug resistance [187]. Interestingly, recent data suggest that FGFR1 inhibitors also decrease resistance to radiotherapy, a widespread problem in glioblastoma [188].

## FGFs in Psychiatric Disorders

Over the years, it has become clear that FGF2 may act as key factor in neuropsychiatric syndromes via activation of FGFR1 [3, 189–191]. The proposed functions range from memory enhancing to anti-depressant and anxiolytic functions. In fact, FGF2 is down-regulated in rats showing high spontaneous anxiety. Knockdown of hippocampal FGF2 activity increases anxiety in naïve rats, and FGF2 treatment reduces anxiety in highly anxious rats [192]. However, mice with genetic ablation of FGF2 isoforms do not show alterations of anxiety-like stress susceptibility [193]. It should be noted, however, that other growth factors (BDNF and IGF-1) exert similar neuromodulatory effects as FGF2 on anxiety-related behaviour, as do genetic and environmental factors.

Traditional anxiolytics interfere with long-term extinction of fear memories. By contrast, chronic extinction is augmented by FGF2 that reduces the likelihood of exhibiting a relapse of extinguished fear in a new environment or following stress. Interestingly, early life treatment with FGF2 may decrease anxiety-like behaviour in adulthood, which probably involves an interaction between FGF receptors and adenosine A2 receptors or dopamine D2 receptors [194]. Furthermore, epigenetic mechanisms may be involved, such as regulation of non-coding RNAs, histone modifications, or DNA methylation [3]. FGF2 promotes the association of trimethylated histone protein H3 at lysine 9 (H3K9me3) at its own promoter [195]. Commonly used bipolar disorder drugs, such as valproate and lithium chloride, have been shown to inhibit histone deacetylases (HDACs) and to increase FGF1 expression [196].

Changes in FGFR levels and genetic variations of FGFR2 have been implicated in the pathomechanisms of schizophrenia [197]. Serum levels of FGF2 are increased in medicated schizophrenia patients and in non-medicated patients exhibiting negative symptoms [198]. FGF signalling disturbances in mood disorders might be indirect, since the disruption of schizophrenia-associated proteins, such as the neuronal PAS domain protein 3 (NPAS3), correlates with a dramatic reduction in FGFR1 mRNA, and NPAS3 deficiency behaviourally resembles FGFR1 knock-out mice [199]. Moreover, the phenotype of FGFR-deficient mice is reminiscent of other animal models for schizophrenia and mood disorders, for example, of knock-outs for disruption in schizophrenia 1 (DISC1), BDNF, and NRG1 [191]. Interestingly, nuclear FGFR1 has been implicated in the disease pathogenesis of schizophrenia. Neuron-committed cells from patients overexpressing FGFR1 reveal an association of nuclear FGFR1 with a large number of genes dysregulated in schizophrenia [27].

FGF signalling is clearly disturbed in individuals with major depressive disorder (MDD). Levels of FGF1, FGF2, FGFR2, and FGFR3 decrease in cortical areas, while FGF9 and FGF12 are elevated in the anterior cingulate and dorsolateral prefrontal cortex of depressive patients [200]. Ubiquitously found NG2 glial cells (precursors for myelinating oligodendrocytes) have been shown to secrete FGF2 during chronic stress, which may prevent glutamate abnormalities and maladaptive depressive behaviour [201]. Direct relationships were suggested when the intracerebroventricular injection of FGF2 resulted in antidepressant effects, which were also observed after FGF2 infusion into the prefrontal cortex in chronic unpredictable stress models of depression [202]. Notably, FGF2 may have indirectly enhanced neuronal activity by the stimulation of astrocyte proliferation, which is reduced in rodent depression models.

Elevated FGF2 levels may also play a role in the therapeutic effects of tricyclic antidepressants and selective serotonin re-uptake inhibitors [203]. The low and high molecular weight isoforms of FGF2 increase in response to monoamine oxidase inhibitor treatment in cortical astrocytes [204], and anti-depressive treatments in rodents elevated FGF2 expression in the hippocampus and cerebral cortex [203, 205]. Inflammatory reactions such as microglia activation and proliferation, which accompany depressive-like behaviours in LPS models, are ameliorated by neuron-derived endogenous FGF2 [171] or by FGF2 infusions [206]. FGF22 is putatively also involved in regulating affective behaviour. FGF22 knock-out mice exhibit depressive symptoms such as longer duration of floating, increased immobility in the tail suspension test, and a decreased preference for sucrose [207].

In animal models of drug use and addiction, FGF2 expression is increased in reward-related infralimbic/medial prefrontal cortex areas, and its neutralisation facilitates extinction of cocaine seeking [208]. Cocaine blocks the re-uptake of dopamine, and the auto-oxidation of dopamine results in free-radicals as by-products [209], which in turn increase FGF2 expression in astrocytes [30]. FGF2 is required for amphetamine-induced sensitisation [210], dendritic growth in dopaminergic neurons, and reductions in intrinsic excitability [211]. These effects are probably mediated not only by FGF2 but also by a cocktail of growth factors that arbitrate maladaptive stimulant induced alterations in neuronal function and structure.

## Therapeutic Approaches

FGFs display a poor blood-brain barrier penetration and have a short half-life. They are vulnerable to proteolytic cleavage events resulting in their inactivation in various body fluids. A recent study demonstrated that a protein delivery coacervate can be prepared that controls FGF2 release and maintains its bioactivity by binding FGF2 via charge interaction consisting of polycation-polyethylene argininylaspartatediglyceride (PEAD) and heparin [212]. Furthermore, biodegradable micro-osmotic pumps based on microelectromechanical system (MEMS) technology have been developed for long-term controlled release of FGF2 [213]. Clearly, novel approaches including small non-peptidergic FGF mimetics will be required for the treatment of central and peripheral nervous system disorders.

Stimulants of FGFR and NCAM, such as the FGF-derived dekafin peptides, will be particularly useful in promoting tissue repair [214, 215]. It will be interesting to determine if these dendrimers might also be beneficial in normalising cognitive and social behaviour by their influence on the excitation/inhibition balance in the brain. Furthermore, FGF-dendrimer-based targeted delivery of drugs through FGFR may be a useful technology to target tumour cells or other FGFR expressing cells [216]. Novel biomaterials such as nontoxic and chemically inert hydrogels provide ideal scaffolds for the ingrowth of regenerating axons. Recently, FGF2 containing HEMA-MOETACL hydrogels were demonstrated to deliver FGF to the injured spinal cord in a localised and sustained manner [217]. Two months after implantation, the hydrogel was surrounded by an acellular vascular matrix consisting of glycosaminoglycan (GAG) and elastic/collagen fibres that promoted FGF-enhanced adhesion and migration of various cells types, resulting in nervous tissue regeneration and functional recovery in the paralysed hindlimbs of rats with complete spinal cord injury.

More recent developments include genetic approaches, for example, neuronal or glial FGF/FGFR transfer via lentiviral vectors (LVs). Gene therapy provides a useful tool for the specific down-regulation or knock-out of neuronal targets, since efficient siRNA treatments and viral gene transfer of shRNAs are now available for humans. Gene replacement therapy has been demonstrated to promote survival of patients with spinal muscular atrophy (SMA) following a single intravenous infusion of adeno-associated virus 9 (AAV9) containing cDNA coding for SMN [218]. LVs are even more useful for *in vivo* applications than AAVs because of their efficiency in gene delivery and their excellent safety profile. Moreover, in contrast with retroviral vectors, LVs do not depend on active division of the cell to be transduced and produce only minimal alteration in cellular physiology [219]. Alternatively, organically modified silica (ORMOSIL) nanoparticles may be used as nonviral vectors for efficient *in vivo* gene delivery. Nucleus-targeting FGFR1 was successfully transfected with this method [220].

## Conclusions

The intricate relationship between oligodendrocytes and Schwann cells and central and peripheral axons, respectively, as well as the close association of ramified astrocytic or microglial processes with neuronal perikarya, neurites, and synapses represent the morphological basis for all FGFR-dependent signalling processes in neuronal and glial cells. More than 10 members of the FGF family function in these spatio-temporal domains, including membrane and nuclear compartments, to regulate transcriptional, post-transcriptional, and post-translational molecular events underlying instant changes in behaviour, long-lasting tissue repair, and axon regeneration. The effects of FGFs are clearly different from other neuronal growth factor families that act in the developing and adult brain. First, they strongly involve glial elements, and, second, they interact with several unrelated receptors via direct physical interaction or formation of heteromeric receptor complexes.

Currently, it is not possible to conclude with certainty whether the neurochemical and morphological alterations induced by FGFR signalling represent causes, consequences, epiphenomena, or a combination of those in the various pathological processes discussed here. Therefore, it is often not clear whether modulators of FGFRs would result in successful treatment that interferes with disease defining pathomechanisms. They could also alleviate symptoms or modify secondary, perhaps even beneficial aspects of brain disorders as exemplified by the negative effects of endogenous FGF2 in experimental models of amyotrophic lateral sclerosis [75]. Hence, the specific cellular context of a given pathology involving primarily glial and/or neuronal mechanisms will decide whether therapeutic interference with FGFRs is indicated or not.

## Data Availability

Not applicable
